# Chromosome alignment and Kif18A action rely on spindle-localized control of Cdk1 activity

**DOI:** 10.3389/fcell.2024.1490781

**Published:** 2024-11-14

**Authors:** Angela Flavia Serpico, Caterina Pisauro, Asia Trano, Domenico Grieco

**Affiliations:** ^1^ Dipartimento di Medicina Molecolare e Biotecnologie Mediche (DMMBM), University of Naples “Federico II”, Naples, Italy; ^2^ CEINGE Biotecnologie Avanzate “Franco Salvatore”, Naples, Italy

**Keywords:** chromosome alignment, i-Cdk1, micronuclei, aneuploidy, Wee1, spindle assembly, cancer vulnerability

## Abstract

**Introduction:**

During mitosis, chromosome alignment at the mitotic spindle equator grants correct chromosome segregation and proper nuclei formation in daughter cells. The kinesin 8 family member Kif18A plays a crucial role for chromosome alignment by localizing at the kinetochore-microtubule (K-MT) plus ends to dampen MT dynamics and stabilize K-MT attachments. Kif18A action is directly antagonized by the master mitotic kinase cyclin B-dependent kinase 1 (Cdk1) and is promoted by protein phosphatase 1 (PP1). Since chromosome alignment precedes Cdk1 inactivation by cyclin B proteolysis, it is unclear how Kif18A evades Cdk1 inhibition.

**Methods:**

We analyzed chromosome alignment and Kif18A in mitotic cells upon genetic perturbation of the phosphorylation-dependent inhibitory control of Cdk1 activity by immunofluorescence and cell fractionation experiments.

**Results:**

We show here that chromosome alignment in human cells relies on a recently identified fraction of Cdk1 that is inhibited by Wee1-dependent phosphorylation in mitosis (i-Cdk1, standing for inhibited/inactive-Cdk1) and that localized at spindle structures where it promotes proper spindle assembly. Indeed, the reduction of i-Cdk1 led to several spindle defects including spindles with misaligned, bipolarly attached chromosomes showing poor Kif18A localization at their K-MT plus ends. Restoring i-Cdk1 reversed both alignment defects and Kif18A localization. In cells with lowered i-Cdk1, expressing a phosphonull Kif18A mutant version at the sites that serve as Cdk1 substrate significantly rescued the alignment defects.

**Discussion:**

Mechanistically, our evidence suggests that i-Cdk1 and active PP1 facilitated the dephosphorylation and reactivation of spindle-localized Kif18A. Considering the relevance of Kif18A for survival of aneuploid cancer cells and the potential therapeutic targeting of both Kif18A and Wee1, these findings could also be relevant for cancer therapy.

## Introduction

During mitosis, proper chromosome alignment at the equator of the mitotic spindle at metaphase grants synchronous chromosome segregation in anaphase, helping to prevent mis-segregation and formation of micronuclei into daughter cells (Risteski et al., 2021; Gomes et al., 2022; Sepaniac et al., 2021). During spindle assembly, chromosomes congress towards the spindle equator in an oscillating fashion, promoted by the action of kinesins and the control of MT dynamics. To reach the metaphase configuration, chromosomes are pushed away from centrosomes by polar ejection forces, mainly acting on chromosome arms, and pulled towards centrosomes by kinetochore-dependent forces, until the final alignment at the center of the spindle (Risteski et al., 2021). This oscillatory behavior is also supposed to facilitate correct chromosome attachments, while the final alignment at the spindle equator is reached by the progressive decline of these oscillatory movements. A central action to dampen chromosome oscillations and promote alignment is exerted by localization of the plus end-directed kinesin 8 family member Kif18A at the plus ends of K-MTs, where it suppresses MT dynamics (Risteski et al., 2021; Gomes et al., 2022; Sepaniac et al., 2021; Mayr et al., 2007; Stumpff et al., 2008; Du et al., 2010). Kif18A concentration at K-MT plus ends has also been involved in the tension-dependent mechanisms that silence the Spindle Assembly Checkpoint (SAC), the safeguard mechanism that prevents anaphase onset until correct spindle assembly completion (Janssen et al., 2018). Although loss of Kif18A function causes micronuclei formation, it appears to be rather tolerated by euploid cells and inactivating mutations in Kif18A have been shown to produce micronuclei in mice without leading to cellular transformation or cancer (Gomes et al., 2022; Sepaniac et al., 2021; Janssen et al., 2018). Nevertheless, Kif18A does appear to be essential for survival of aneuploid cancer cells, perhaps by preventing unbearable chromosome mis-segregation in cells with more complicated spindle architecture, and is a vulnerability target for certain aneuploid cancers (Quinton et al., 2021; Cohen-Sharir et al., 2021; Marquis et al., 2021; Payton et al., 2024; Gliech et al., 2024). The ability of Kif18A to reach the K-MT plus ends and dampen chromosome oscillations has been shown to be antagonized by phosphorylation operated by Cdk1 and stimulated by its reversal operated by PP1 (Häfner et al., 2014). How Kif18A evades inhibition by Cdk1 during spindle assembly is unknown. We show here that the ability of Kif18A to reach K-MT plus ends and promote chromosome alignment depends on i-Cdk1, a spindle-localized fraction of Cdk1 that we have recently described to be inhibited by phosphorylation in mitosis and to be crucial for spindle assembly (Serpico et al., 2022; Serpico et al., 2023).

## Materials and methods

### Cell lines and cell culture

Human Henrietta Lacks (HeLa; CEINGE Cell Culture Facility) cells were grown in Dulbecco’s Modified Eagle Medium high glucose (DMEM; Sigma-Aldrich) supplemented with 10% foetal bovine serum (FBS; Gibco), 1% GlutaMAX-supplement (Gibco), 1% penicillin/streptomycin (Euroclone). Human Telomerase Reverse Transcriptase-immortalized Retinal Pigment Epithelial (hTERT-RPE1; Dr. A. Musacchio’s gift; Max Planck Institute of Molecular Physiology, Dortmund) cells were grown in Dulbecco’s Modified Eagle Medium: Nutrient Mixture F-12 (DMEM/F12; Gibco, Thermo Fisher Scientific) supplemented with 10% foetal bovine serum (FBS; Gibco), 1% GlutaMAX-supplement (Gibco), 1% penicillin/streptomycin (Euroclone). All cells were incubated in a humidified incubator at 37°C with 5% CO_2_.

### DNA and siRNA constructs

Cdk1-WT and Cdk1-AF mammalian expression plasmids were purchased from Addgene (Cat# 61840; Cat# 39872, respectively). 3XFlag-Wee1 expression construct was obtained as previously described (Visconti et al., 2015). Myc-Flag-tagged-human-Kif18A-WT (Kif18A-WT) plasmid was purchased from Origene (Cat# RC208235); Kif18A-AA mutant version was generated via mutagenesis: serine 674 was mutagenized into alanine (2020–2022 nt TCT→GCT) and serine 684 into alanine (2050–2052 nt TCT→GCT) by QuikChange II XL site-directed mutagenesis kit (Agilent Technologies) using Kif18A-WT as template. Custom siRNAs targeting the 3^’^-UTR of human WEE1 (#1: 5′-CUG​UAA​ACU​UGU​AGC​AUU​AUU-3’; #2: 5′-GUA​CAU​AGC​UGU​UUG​AAA​UUU-3’; #3: 5′-GGG​CUU​UAU​UAC​AGA​CAU​AUU-3′) were designed using siDESIGN Center tool by Horizon and purchased from Dharmacon.

### Transfection and RNA interference

Cells were seeded at a cell density of 7,000/cm^2^ either into 6–10 cm dishes or onto glass coverslips for biochemical and immunofluorescence studies, respectively. Manufacturer’s protocols were followed for both procedures and cells were plated 24 h prior to treatment. Transient expression transfections were performed using MegaTran 2.0 Transfection Reagent (OriGene; Cat# TT210003); protein downregulation via siRNAs was obtained using DharmaFECT 1 siRNA Transfection Reagent (Dharmacon; Cat# T-2001–03). Briefly, MegaTran (1 μg/μL): DNA (1 μg/μL) (3:1) or DharmaFECT 1: siRNAs duplex (25 nM) (1:1) were mixed in cell culture media (DMEM or DMEM/F12) and incubated at room temperature (rt) for 20 min. Then the mixtures were added to the cells maintained in antibiotic-free complete medium and incubated for 24 h (see also cell synchronization section). For Wee1-siRNA treatment and complementation, cells were transfected with mock- or 3XFlag-Wee1 expression construct 24 h prior to treatment with non-targeting or specific siRNAs and 6 h after siRNA-treatment cells were *ad hoc* synchronized (see also cell synchronization section). Exogenous Cdk1-WT or Cdk1-AF overexpression was achieved by transfection of the respective plasmids (empty or Cdk1-WT or Cdk1-AF expression vectors) 8 h prior to specific synchronization (see also cell synchronization section). For co-overexpression experiments, Cdk1-AF + empty vector, Cdk1-AF + Kif18A-WT and Cdk1-AF + Kif18A-AA vectors were simultaneously transfected 8 h prior to synchronization.

### Cell synchronization

Prometaphase-arrested HeLa cells were obtained by a 14-hour incubation with nocodazole (1 μg/mL; Abcam; Cat# ab120630). G2-arrested cells were obtained by incubation with RO-3306 at 9 μM (Calbiochem; Cat# 217699) for 16 h. To obtain metaphase-arrested cells, G2-or prometaphase-arrested cells were washed twice with fresh medium and twice with phosphate buffer saline 1X (PBS; Corning; Cat# 21–031-CV) solution before plating into fresh medium containing MG-132 (20 μM; Calbiochem; Cat# 474790) and cycloheximide (CHX; 60 μg/mL; Santa Cruz Biotechnology; Cat# sc-3508) for further 60 min of incubation. Where indicated, mild inhibition of Cdk1 activity (+RO) was obtained by adding RO-3306 at 0.5 μM (Calbiochem).

### Cell fractionation

Cell fractionation was performed as previously described (Serpico et al., 2022). To separate cytoplasmic and spindle-bound proteins we adjusted a previously described method (Silljé and Nigg, 2006). Cells were collected by centrifugation and washed once with PBS containing taxol (1μM; Sigma-Aldrich). 5 × 10^6^ cells were carefully resuspended in 800 μL of fractionation lysis buffer (FLB; 40 mM β-glycerophosphate, 15 mM MgCl_2_, 20 mM EGTA, 0.25% Igepal, 20 mM Hepes, 5 μM taxol, 6 μg/mL latrunculin B; Sigma-Aldrich) completed with 300 μg/mL RNase A and 120 U/mL DNase I (Roche) and then incubated in thermomixer (Eppendorf thermomixer comfort) with constant shaking at 1.200 rpm for 20 min at 34°C. Lysates were centrifuged at 6.800 rpm (Eppendorf centrifuge 5,425) for 2 min: the supernatants were harvested into new tubes, supplemented with 250 mM NaCl and stored on ice (soluble fractions). The pellet fractions were once again resuspended in FLB + 300 μg/mL RNase A and 120 U/mL DNase I and incubated in thermomixer at 34°C for 20 min. Samples were centrifuged and pellet fraction were washed twice with washing buffer (WB; 5 mM Hepes, 5 μM taxol; Sigma-Aldrich). Then, pellet fractions were resuspended in 200 μL of FLB minus taxol and supplemented with 250 mM NaCl and incubated on ice for 30 min. Finally, soluble and pellet fractions were spun at 13.200 rpm at 4°C for 10 min (Eppendorf centrifuge 5424 R) and both final supernatants were collected and processed for Immunoblottings (Ibs) or Immunoprecipitations (Ips). The pellet fraction proteins were extracted in a 200 μL buffer volume from a total cell lysate of 800 μL volume (mainly representing the soluble fraction volume), hence pellet fraction proteins were enriched 4 folds over the soluble fraction proteins, relatively to their cellular distribution and, in the experiment shown in Figure 3, a double volume of the pellet relatively to the soluble fractions was loaded on gels, thus, pellet fraction proteins were enriched a total of 8 folds over the soluble fraction proteins.

### Immunoprecipitation

For immunoprecipitations from total cell lysates 5 × 10^6^ cells were lysed in 180 μL lysis buffer (LB; 80 mM β-glycerophosphate, 15 mM MgCl_2_, 20 mM EGTA, 20 mM Hepes pH 7.4, 100 mM NaCl, 0.1% Igepal; Sigma-Aldrich) supplemented with a phosphatase inhibitor cocktail (PhosSTOP; Roche) and incubated on ice for 30 min. Then, lysates were cleared via centrifugation at 13.200 rpm at 4°C for 10 min (Eppendorf centrifuge 5424 R) and incubated with agarose bead-conjugated antibody overnight at 4°C in constant rotation (mouse anti-cyclin B1 agarose, Santa Cruz Biotechnology, Cat# sc-245AC; normal mouse IgG-AC, Santa Cruz Biotechnology, Cat# sc-2343). For immunoprecipitations from soluble and pellet fractions, lysates were diluted in LB supplemented with PhosSTOP (Roche) until a final volume of 800 μL and incubated with agarose conjugated antibodies overnight at 4°C in constant rotation. For both above-mentioned cases, beads were washed twice in LB and proteins eluted in Laemmli denaturing buffer by boiling for 5 min (Laemmli sample buffer; BioRad, Cat# 1610747). Finally, samples were loaded into polyacrylamide gels and analyzed by immunoblotting.

### Immunoblotting

Immunoblotting was performed as described (Serpico et al., 2022). Briefly, samples in SDS Laemmli buffer were incubated for 10 min at 99°C and then loaded and run on SDS-PAGE gels (polyacrylamide percentage spanning from 10% to 12%; differently migrating forms of Kif18A were resolved on 10% polyacrylamide gels). Proteins were transferred onto nitrocellulose membrane (Cytiva-Amersham; Cat# GEH10600002) using a wet-transfer system (Thermofisher) and membranes were blocked with 5% nonfat dry milk (NFDM; AppliChem; Cat# A0830) in PBS supplemented with 0.01% Tween20 (TPBS; Sigma-Aldrich; Cat# P1379) for 1 h at rt. Then, filters were incubated with primary antibodies, diluted in TPBS, at 4°C overnight. After 2 TPBS washes, membranes were incubated with secondary peroxidase-conjugated (HRP) antibodies, also diluted in TPBS, for 1 h at rt. Enhanced ChemiLuminescence (ECL) kit (Cytiva-Amersham; Cat# RPN2106) enabled the detection of HRP enzyme activity. Blots were acquired using Canon CanoScan LiDE 300 scanner (Canon) and scanned at 300 dpi. For western blot analysis, primary antibodies were used as follows: rabbit anti-Wee1 (1:1000; Cell Signaling Technology; Cat# 13084); rabbit anti-cyclin B1 (1:2000; Bethyl; Cat# A305-000A); mouse anti-cdc2 (1:500; BD Biosciences; Cat# 610038); rabbit anti-Kif18A (1:1000; Bethyl; Cat# A301-080A); mouse anti-γ-tubulin (1:2000; Sigma-Aldrich; Clone GTU-88; Cat# T5326), rabbit anti-phospho-tyrosine-15-cdc2 (1:1000; p-Y15-cdc2; Cell Signaling Technology, Cat# 4539), rabbit anti-phospho-Threonine-320-PP1α (1:1000; p-T320-PP1α; 1:1000; Cell Signaling Technology; Cat# 2581), mouse anti-PP1α (1:1000; Santa Cruz Biotechnology, Cat# sc-7482). Sheep anti-mouse IgG HRP linked (1:2000; GE Healthcare; Cat# NA931), donkey anti-rabbit IgG HRP linked (1:2000; GE Healthcare; Cat# NA934) were used as secondary antibodies. Band intensity was quantified by measuring the optical density (OD) of a fixed area surrounding bands, applied to all lanes, using the ImageJ software, subtracting background (expressed as arbitrary units).

### Immunofluorescence and microscopy

Cells were plated onto poly-D-lysine (0.1 mg/mL; Sigma-Aldrich; Cat# P6407) coated glass coverslips at a cell density of 7,000/cm^2^. After a brief wash in PBS, cells were fixed and permeabilized with 4% paraformaldehyde +0.5% Triton X-100 (Sigma-Aldrich; Cat# P6148; T9284 respectively) in PBS for 12 min at rt. Then, cells were washed twice with PBS and incubated with 1.5% bovine serum albumin (BSA; Sigma-Aldrich; Cat# A7030) in PBS for 1 h at rt. After 2 PBS washes, cells were incubated with primary antibodies in 1.5% (w/v) BSA-PBS solution for 3 h into a humidity chamber at rt. Afterwards, cells were washed 3 times with PBS and incubated with fluorescently labelled secondary antibodies solution (1.5% BSA-PBS) for 1 h at rt. DNA was stained with Hoechst 33,258 (1 μg/mL; Invitrogen; Cat# 94403) by incubation for 10 min. Finally, cells were washed 4 times with PBS and slides mounted with Mowiol 40–88 (Sigma Aldrich; Cat# 81381). For immunofluorescence staining, the following antibodies were used: mouse anti-α-tubulin (1:1000; Sigma-Aldrich; Clone DM1A; Cat# T9026); human anti-centromere (CREST; 1:100; Antibodies Incorporated; Cat# 15–234); rabbit anti-Kif18A (1:2000; Abcam; Cat# ab251863); donkey anti-mouse IgG Alexa Fluor 594 (1:1000; Invitrogen; Cat# A21203); goat anti-human IgG Alexa Fluor 488 (1:1000; Invitrogen; Cat# A11013); donkey anti-rabbit IgG Alexa Fluor 594 (1:1000; Invitrogen; Cat# A21207). Inverted confocal fluorescence microscope LSM 980 (Zeiss) equipped with a 63X/1.4 oil objective (Zeiss) was used to image fixed cells, image acquisition settings were identical for all samples. Three image planes with 0.44 mm Z-stack size were acquired and projected into one plane using ZEN3.1 software (Zeiss).

### Quantitative analyses of chromosome alignment, spindle length and Kif18A and α-tubulin fluorescence intensity

Spindles were scored as with misaligned chromosomes when more than three chromosomes (three centromere pairs; scored by CREST staining) were outside the two internal quarters of the interpolar distance. Around 100 cells were scored in 4 independent microscopy slide fields per sample in at least three independent experiments performed under similar experimental conditions. Average spindle length was scored by measuring the distance (μm) from the two opposite α-tubulin apexes in a total of 45 bipolar spindles per condition from three independent experiments (15 spindles per condition for each experiment). Statistical analyses and p-value assessments through a two-tailed unpaired *t*-test were performed using GraphPad software. For quantitation of Kif18A and α-tubulin immunofluorescence signals, cell staining conditions and image acquisition settings were identical for all samples and the mean fluorescence intensity of the spindle area was quantified from 20 bipolar spindles per condition from 2 independent experiments (total of 40 spindles analyzed per condition) through the ImageJ software and expressed as arbitrary units. Statistical analyses and p-value assessments were obtained through a two-tailed unpaired *t*-test performed using the GraphPad software.

## Results

### Dependence of chromosome alignment on inhibitory control of Cdk1

**FIGURE 1:**
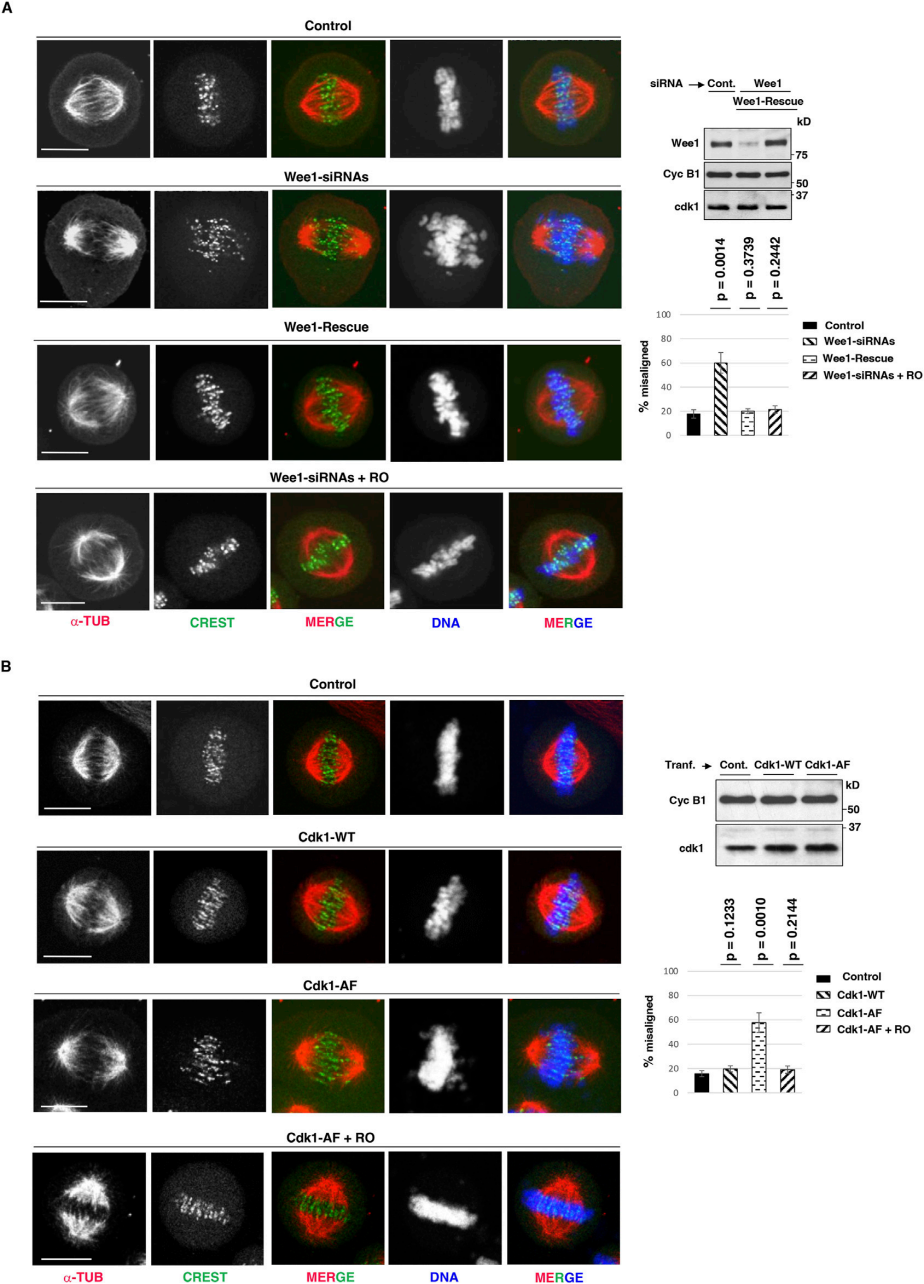
Dependence of chromosome alignment on i-Cdk1. **(A)** HeLa cells were treated with: non-targeting siRNAs (Control), Wee1-targeting siRNAs (Wee1-siRNAs) and Wee1-targeting siRNAs in cells previously transfected with siRNA-resistant Flag-tagged-Wee1 expression vector (Wee1-Rescue). Cells were arrested at G2, by a 16-hour treatment with high concentrations of the selective and reversible inhibitor RO-3306 (9 μM; added 6 h post siRNA-treatments), released into fresh MC medium (see Material and Methods section), fixed after 80 min of further incubation and analyzed by immunofluorescence. To a sample of Wee1-siRNAs, low concentrations of RO-3306 (0.5 μM) were added by 60 min from G2-arrest release (Wee1-siRNAs + RO). Immunofluorescence images: cells were stained for CREST (centromere marker, green), α-tubulin (α-TUB, red) and DNA (blue). Addition of vehicle (DMSO) had no effect on spindle assembly in Wee1-siRNA-treated cells. Scale bar: 10 μm. Graph: percent of bipolar spindles with misaligned chromosomes (more than three chromosomes falling outside the two internal quarters of the interpolar distance) in Control, Wee1-siRNAs, Wee1-Rescue and Wee1-siRNAs + RO cells. Around 100 cells were scored in 4 independent microscopy slide fields per sample. Error bars refer to standard deviation of percent of bipolar spindles with misaligned chromosomes in three independent experiments performed under similar experimental conditions. The p-values, reported above the bars, were calculated from comparison of percent of bipolar spindles with misaligned chromosomes of Control cells with that of Wee1-siRNA cells (*p* = 0.0014), Wee1-Rescue cells (*p* = 0.3739), and Wee1-siRNAs + RO cells (*p* = 0.2442) in three independent experiments, using a two-tailed unpaired *t*-test. Blots: cell samples from Control (non-targeting; Cont.), Wee1-siRNAs, Wee1-Rescue were lysed and lysates probed for the indicated antigens. **(B)** HeLa cells were transfected with an empty vector (Control), with a wild type Cdk1 (Cdk1-WT) expression vector or with a mutant Cdk1 expression vector in which Cdk1-threonine 14 and tyrosine 15 are mutated into non-phosphorylatable alanine and phenylalanine, respectively (Cdk1-AF). 6 h post transfection, cells were arrested at G2 by addition of RO-3306 (9 μM) for further 16 h of incubation, released into fresh MC medium (see Material and Methods section), fixed after 80 min of further incubation and analyzed by immunofluorescence. Cells were stained for DNA (blue), α-tubulin (α-TUB, red) and CREST (green) at 80 min upon release from G2 arrest. A portion of Cdk1-AF-transfected cells received low-RO at 60 min post release from G2-arrest (Cdk1-AF + RO); as control vehicle was added (DMSO). Scale bar: 10 μm. Graph: percent of bipolar spindles with misaligned chromosomes in Control, Cdk1-WT, Cdk1-AF, and Cdk1-AF + RO cells. Spindles were scored as with misaligned chromosomes when more than three chromosomes were outside the two internal quarters of the interpolar distance. Around 100 cells were scored in 4 independent microscopy slide fields per sample. Error bars refer to standard deviation of percent of bipolar spindles with misaligned chromosomes in three independent experiments performed under similar experimental conditions. The p-values, reported above the bars, were calculated from comparison of percent of bipolar spindles with misaligned chromosomes of Control cells with that of Cdk1-WT (*p* = 0.1233), Cdk1-AF (*p* = 0.0010) and Cdk1-AF + RO cells (*p* = 0.2144) in three independent experiments, using a two-tailed unpaired *t*-test. Blots: cell samples from Control, Cdk1-WT and Cdk1-AF were lysed and lysates probed for the indicated antigens.

We have recently gathered evidence that mitotic spindle assembly in human cells relies on the inhibitory control of a small and localized fraction of Cdk1, i-Cdk1, that represents less than 10% of total Cdk1 present in mitotic cells (Serpico et al., 2022; Serpico et al., 2023). While bulk, active, Cdk1 maintains the cytoplasm of mitotic cells substantially free of MTs, i-Cdk1 appears required at spindle structures to locally reactivate MT-stabilizing MT-Associated Proteins (MAPs) to promote spindle assembly (Serpico et al., 2022; Serpico et al., 2023). In experiments in which i-Cdk1 was lowered in mitotic cells either by downregulation of Cdk1 inhibitory kinase Wee1, by small interfering RNAs (siRNAs), or by overexpression of an inhibitory phosphorylation-resistant Cdk1 version at the inhibitory sites threonine 14 and tyrosine 15 (Cdk1-AF), we observed severe alterations in spindle assembly relatively to control cells, ranging from monopolar spindles to bipolar spindles with deranged microtubular structures and chromosome alignment (Serpico et al., 2022). To further study the alignment defects caused by i-Cdk1 loss, we analyzed in more details HeLa cells that assembled bipolar spindles upon Wee1 downregulation or Cdk1-AF overexpression (Figures 1A, B) (Serpico et al., 2022). HeLa cells treated with non-targeting (NT) siRNAs, as control, or with siRNAs targeting Wee1 (Wee1-siRNAs) were arrested at G2, by a 16-h treatment with the selective and reversible inhibitor RO-3306 (9 μM), released into fresh medium, containing the proteasome inhibitor MG-132 and the protein synthesis inhibitor cycloheximide, to block mitosis exit and prevent excess protein accumulation (MC medium), fixed after 80 min of further incubation and analyzed by immunofluorescence (IF; Figure 1A) (Vassilev, 2006). Part of Wee1-siRNAs cells were also complemented with a siRNA-resistant Wee1 expression vector (Wee1-Rescue; Figure 1A) (Visconti et al., 2015). As previously shown under similar experimental conditions, the majority (around 80%) of control NT-siRNA- or Wee1-Rescue cells were able to build normal bipolar spindles within 60 min of incubation, that remained stable for at least further 20 min incubation, while spindle assembly was substantially impaired in the majority of Wee1-siRNAs cells (Serpico et al., 2022). Nevertheless, about 40% of Wee1-siRNAs cells could mount bipolar spindles but spindles showed alignment defects with bioriented chromosome pairs that failed to align at the equator of the metaphase plate in the majority of these cells (Figure 1A) (Serpico et al., 2022). In these cells, spindles appeared also elongated, showing a higher average spindle length compared to the control and Wee1-Rescue cells, respectively (Supplementary Figure S1A). Similar defects in bipolar spindles were also observed by overexpressing Cdk1-AF, but not by wild type Cdk1 (Cdk1-WT), in HeLa cells (Figure 1B; Supplementary Figure S1B) and in the non-transformed hTERT-RPE1 cells (Supplementary Figure S2A, B). Importantly, mild Cdk1 inhibition in both Wee1-siRNAs and Cdk1-AF-overexpressing cells, by addition of low concentrations of RO-3306 (0.5 μM; + RO) from 60 to 80 min post G2 release, substantially reversed spindle defects compacting spindles and inducing tight chromosome alignments (Figure 1A, B; see also Supplementary Figures S1A, B, S2 A, B) (Serpico et al., 2022).

### I-Cdk1 drives K-MT plus end Kif18A accumulation

**FIGURE 2:**
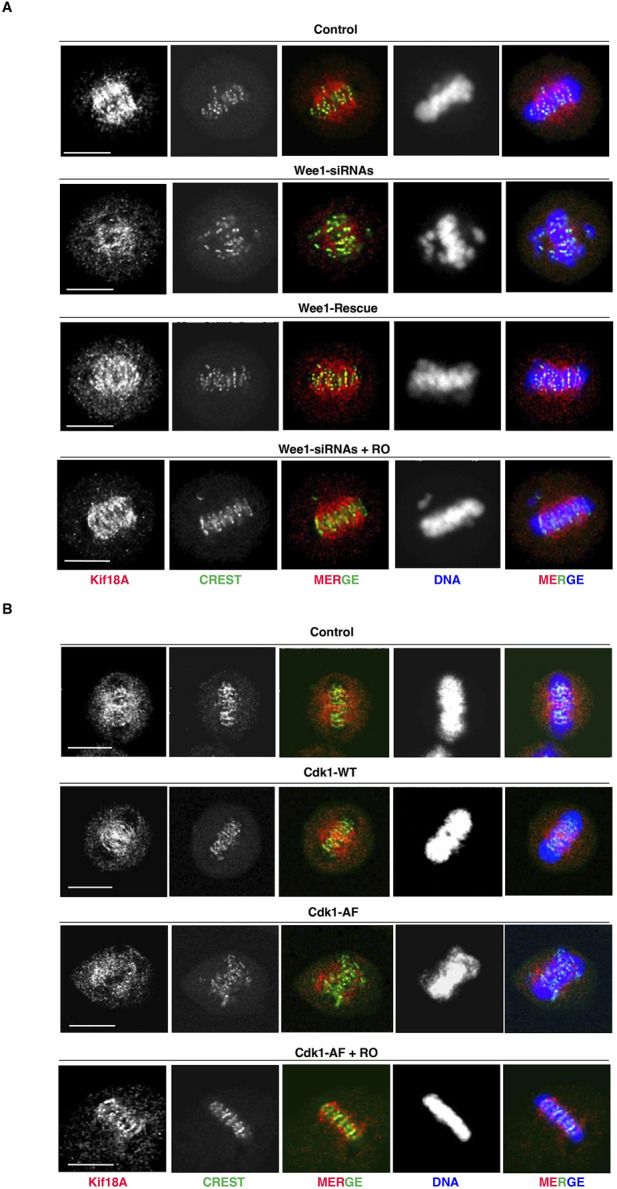
Kif18A accumulation at K-MT plus ends and i-Cdk1. **(A)** HeLa cells were treated as described in Figure 1A and analyzed by immunofluorescence by 80 min of further incubation after release from G2-arrest into fresh MC medium. Kif18A (red), CREST (green), and DNA (blue). Scale bar: 10 μm. **(B)** HeLa cells were treated as described in Figure 1B and analyzed by immunofluorescence by 80 min of further incubation after release from G2-arrest into fresh MC medium. Kif18A (red), CREST (green), and DNA (blue). Scale bar: 10 μm. For quantitation of percent of bipolar spindles with misaligned chromosomes, refer to graphs shown in Figures 1A, B, respectively.

The alignment of bipolarly attached chromosomes at the spindle equator has been shown to require the MT-plus end-directed kinesin Kif18A that accumulates at the center of the spindle where it dampens K-MT dynamics (Risteski et al., 2021; Mayr et al., 2007; Stumpff et al., 2008; Du et al., 2010). Since the ability of Kif18A to concentrate at the K-MT plus ends and inhibit chromosome oscillations has been shown to be antagonized by direct Cdk1-dependent phosphorylation (Häfner et al., 2014), we asked whether i-Cdk1 was required for K-MT plus end localization of Kif18A on bipolar spindles (Figure 2). Under the same experimental conditions of the experiments described in Figure 1, we found that in control metaphase cells Kif18A concentrated at the center of the spindle, accumulating at the plus ends of K-MT facing centromeres (Figures 2A, B). Conversely, in Wee1-siRNAs or Cdk1-AF-overepressing cells the overall amount of Kif18A on spindles was lower than control cells and in particular it failed to concentrate at the spindle center and at the plus ends of K-MT, rather remaining around the spindle pole area (Figures 2A, B). Mild Cdk1 inhibition, in Wee1-siRNAs or Cdk1-AF-overepressing cells, restored high Kif18A concentrations at the spindle center in close centromere proximity, along with tight chromosome alignments (Figures 2A, B; + RO).

### Kif18A and Cdk1 interaction

**FIGURE 3:**
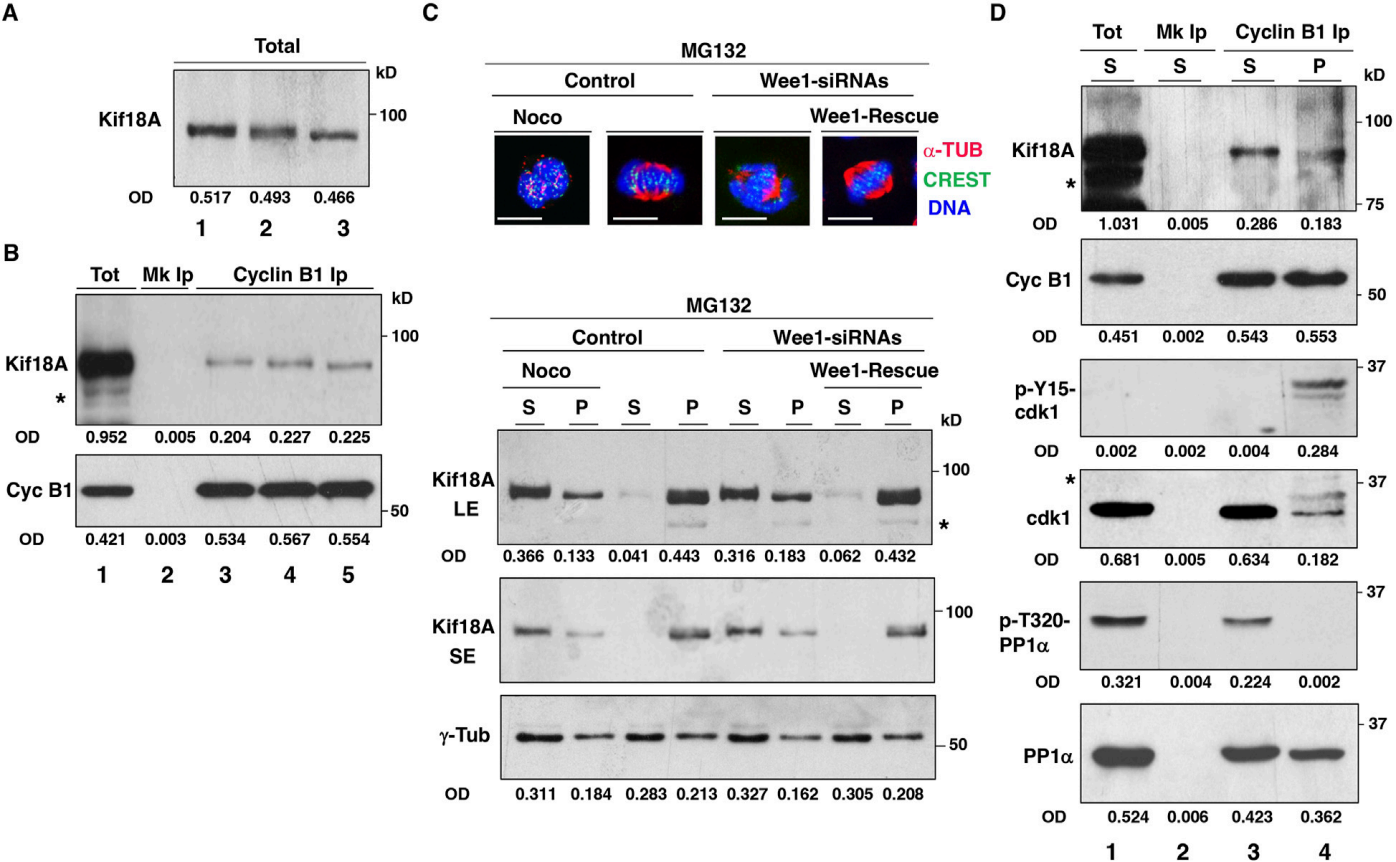
Kif18A and Cdk1 interaction. HeLa cells were arrested at prometaphase by 14 h treatment with nocodazole, released into MC medium and either nocodazole, DMSO or RO3306 (9 μM) was added and incubation prolonged for further 60 min. **(A)** Total cell lysate blots were directly probed for the indicated antigen (lane 1, nocodazole; lane 2, DMSO; lane 3, RO3306). **(B)** Blots of cyclin B1 immunoprecipitates (Ips) from cell lysates were probed for indicated antigens (lane 1: total cell lysate from nocodazole-treated sample; lane 2: mock Ip from nocodazole-treated sample, lane 3, 4 and 5: cyclin B1 Ips from nocodazole, DMSO and RO3306 treated samples, respectively). **(C)** HeLa cells were treated with non-targeting siRNAs (Control), Wee1-targeting siRNAs (Wee1-siRNAs) and Wee1-targeting siRNAs to previously transfected with siRNA-resistant Flag-tagged-Wee1 expression vector (Wee1-Rescue), arrested at prometaphase by 14 h treatment with nocodazole and released into MC medium for 60 min. To a Control sample, nocodazole was added back to keep cells arrested at prometaphase (Noco). Immunofluorescence images: cells were fixed and stained for CREST (green), α-tubulin (α-TUB, red) and DNA (blue). Scale bar: 10 μm. Blots: Cells were fractionated into soluble (S) and pellet (P) fractions that were probed for indicated antigens (for Kif18A short, SE, and long, LE, blot exposures are shown). **(D)** Cyclin B1 Ips from S fraction of Control-Noco-cells (arrested at prometaphase) and from P fraction of Control cells (arrested at metaphase) were probed for indicated antigens (lane 1: total S fraction of Noco cells; lane 2: mock Ip from S fraction of Noco cells, lane 3, and 4: cyclin B1 Ips from S fraction of Control-Noco-cells and from P fraction of Control cells, respectively). OD, optical density of the band area (arbitrary units). Asterisks in Figures 3B, C and D indicate aspecific bands or probable Kif18A degradation products in overexposed blot samples. The data shown are representative of at least three independent experiments performed under similar experimental conditions.

The presence of i-Cdk1 appears required for correct spindle MT polymerization and spindle assembly (Serpico et al., 2022). Thus, the poor localization of Kif18A at the spindle center in cells with reduced i-Cdk1 could be an indirect consequence of the overall impairment of spindle assembly and reduced spindle MT polymerization in those cells, rather than a direct requirement for i-Cdk1 in regulating Kif18A. We co-stained control cells and Wee1-siRNA cells (treated as described for the experiments shown in Figures 1, 2) for Kif18A and α-tubulin and quantitated the mean signal intensity of Kif18A and α-tubulin in the spindle area. Both Kif18A and α-tubulin signal intensity were lower in cells with reduced i-Cdk1 compared to control cells (Supplementary Figure S3). However, also the ratio of Kif18A/α-tubulin signal intensity was sligthely but significantly lower in cells with reduced i-Cdk1 compared to control suggesting that, in addition to spindle MT polymerization, i-Cdk1 may also directly affect Kif18A localization (Supplementary Figure S3). Moreover, the function of Kif18A has been demonstrated to be hindered by direct phosphorylation of Kif18A by Cdk1 during mitosis (Häfner et al., 2014). Analysis of metaphase-arrested cell lysates on SDS/PAGE has revealed that Kif18A exist in fast and slow migrating forms, indicating different phosphorylation states (Häfner et al., 2014). Furthermore, the slow migrating form of Kif18A was converted into the fast migrating form when mitotic cells were treated with a chemical Cdk1 inhibitor, suggesting that it was phosphorylated by Cdk1 (Häfner et al., 2014). In our SDS/PAGE analysis, we did not observe distinct variations in the migration patterns of Kif18A in total mitotic cell lysates. However, we did notice that the band appeared wider in metaphase-arrested cells compared to prometaphase-arrested cells, indicating potential dephosphorylation of Kif18A from prometaphase to metaphase (Figure 3A; lanes 1 and 2). However, we also observed a clear conversion of Kif18A into a faster migrating form after treatment of metaphase-arrested cells with a chemical Cdk1 inhibitor, as reported by Häfner et al. (Figure 3A; lane 3) (Häfner et al., 2014). In order to better understand the connection between Cdk1 and Kif18A, we initially investigated whether Cdk1 and Kif18A physically interacted in mitotic cells by probing cyclin B1 co-immunoprecipitations from total cell extracts (Figure 3B). We found that Kif18A co-immunoprecipitated with cyclin B1 in both prometaphase and at metaphase, even after treatment with the Cdk1 inhibitor (in the presence of the proteasome inhibitor MG132; Figure 3B). Next, we analyzed Kif18A distribution upon fractionating mitotic cells into insoluble pellet fraction (P), containing spindles and spindle-associated proteins, and soluble fraction (S), to further investigate the relationship between Kif18A and Cdk1 (Figure 3C) (Silljé and Nigg, 2006). Control, Wee1-siRNAs and Wee1-Rescue cells were prometaphase-arrested by a 14-hour treament with the reversible microtubule inhibitor nocodazole, added shortly after siRNA treatments, and released into MC medium for further 60 min of incubation (Figure 3C). To a portion of control cells, nocodazole was added back at the beginning of the 60 min incubation to block spindle assembly and keep cells arrested at prometaphase (Figure 3C; Noco). After nocodazole wash out, the majority of control and Wee1-Rescue cells built spindles during the 60 min incubation and arrested at metaphase (Figure 3C; top images). Conversely, as previously shown, spindle formation in Wee1-siRNAs cells was severely impaired (Figure 3C; top images) (Serpico et al., 2022). Following cells fractionation, Kif18A was examined in both soluble and pellet fractions (Figure 3C; bottom blots; pellet protein samples were enriched eight times relatively to soluble protein samples; see Material and Methods section for details).While Kif18A was more abundant in the soluble rather than in the pellet fraction in control prometaphase-arrested cells (Figure 3C; bottom blots; Noco), in control metaphase cells significant Kif18A amounts were recovered in the pellet rather than in the soluble fraction (Figure 3C; bottom blots). Kif18A distribution between soluble and pellet fractions in Wee1-siRNAs cells was similar to prometaphase cells, conversely, in Wee1-Rescue cells the distribution was similar to control metaphase cells (Figure 3C; Wee1-siRNAs and Wee1-Rescue; bottom blots). Blots were also probed for γ-tubulin as control because centrosomes, that are enriched in γ-tubulin, did pelleted even from prometaphase cells and the relative γ-tubulin content in pellet and soluble fractions did not vary substantially between prometaphase and metaphase cells, as previously demonstrated (Figure 3C; bottom blots) (Serpico et al., 2022). Kif18A from the pellet fractions of Control and Wee1-Rescue cells resolved into two differently migrating forms of which the faster form was predominant over the slower migrating form, analyzing short exposures of the Kif18A blots (Figure 3C; Kif18A SE; bottom blots). This observation suggests that Kif18A was present on spindles in a dephosphorylated state. We further analyzed the Kif18A interaction with Cdk1 in the soluble and pellet fractions of prometaphase- and metaphase-arrested cells, respectively, by cyclin B1 co-immunoprecipitations (Figure 3D; asterisks in Figures 3B–D indicate aspecific bands or probable Kif18A degradation products in overexposed blot samples). To this end, we immunoprecipitated comparable amounts of cyclin B1 from the prometaphase-arrested cells’ soluble fraction (Figure 3D; lane 3) and from the metaphase-arrested cells’ pellet fraction (Figure 3D; lane 4). In contrast to Kif18A co-precipitating with cyclin B1 from the soluble fraction of prometaphase-arrested cells, Kif18A co-precipitating with cyclin B1 from the pellet fraction of metaphase-arrested cells migrated faster on SDS/PAGE, indicating its dephosphorylated state (Figure 3D). Furthermore, as previously demonstrated, cyclin B1 immunoprecipitated from the soluble fraction of prometaphase-arrested cells was in an active Cdk1 complex bacause it bound to Cdk1 non-phosphorylated at inhibitory Y-15-Cdk1 site and to inactive PP1α, phosphorylated at the inhibitory T-320 (Figure 3D) (Serpico et al., 2022). On the other hand, in the pellet fraction of metaphase-arrested cells, cyclin B1 bound to Cdk1 that was substantially phosphorylated at the inhibitory Y-15 site and to potentially active PP1α, dephosphorylated at the inhibitory T-320, thus, it was primarely in an i-Cdk1 complex (Figure 3D) (Serpico et al., 2022). Together, these data strongly suggest that i-Cdk1 promoted localized PP1-dependent Kif18A dephosphorylation and activation at spindle MTs.

### Alignment defects rescue by expression of a Cdk1-phosphorylation-resistant Kif18A mutant in i-Cdk1-downregulated cells

**FIGURE 4:**
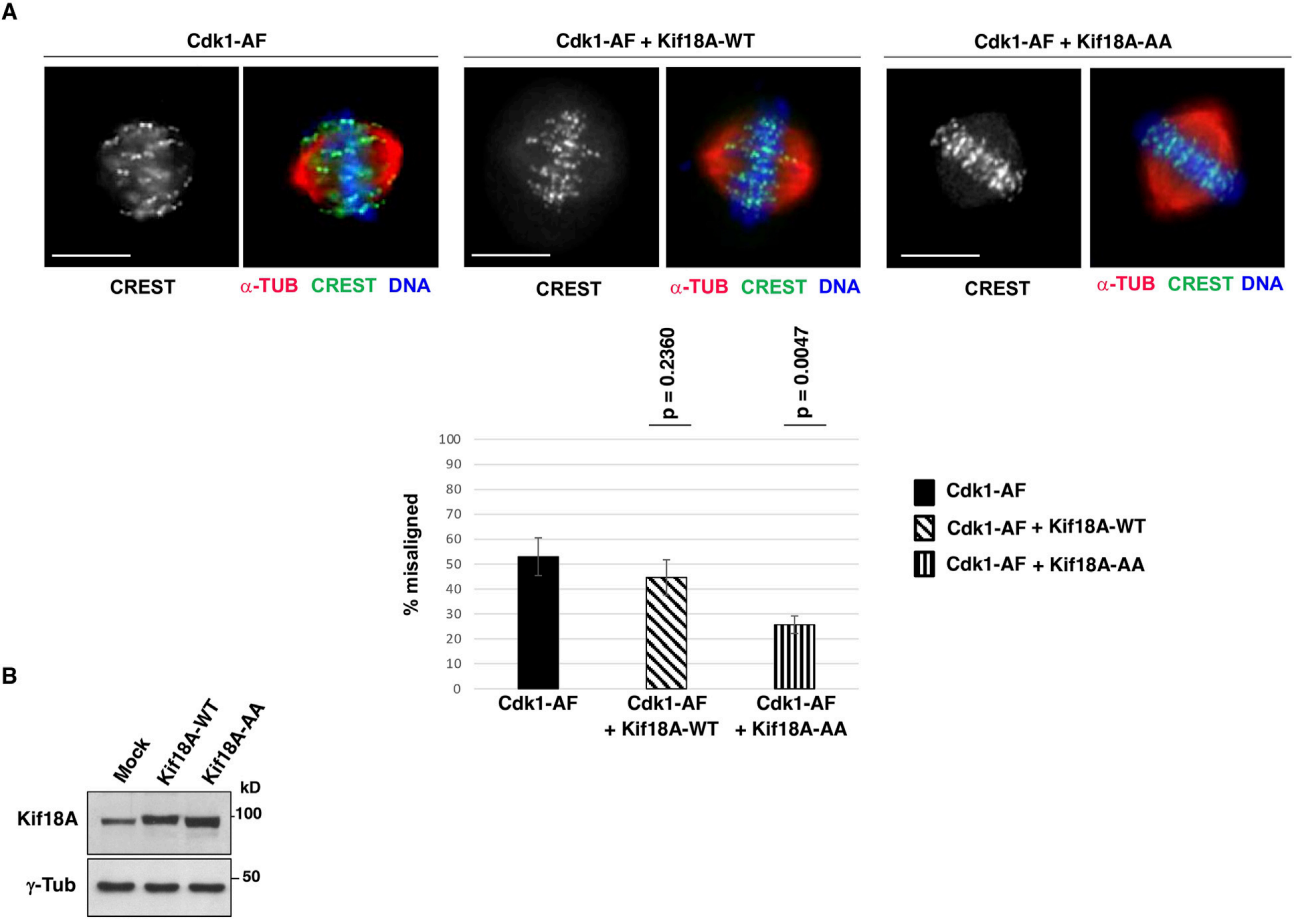
Cdk1-phosphorylation-resistant Kif18A mutant expression improves alignment in i-Cdk1-downregulated cells. **(A)** HeLa cells were co-transfected with a Cdk1-AF expression vector plus an empty vector (Cdk1-AF), with a Cdk1-AF expression vector plus a wild type Kif18A-expression vector (Cdk1-AF + Kif18A-WT) or with a Cdk1-AF expression vector plus a mutant S674A/S684A-Kif18A-expression vector (Cdk1-AF + Kif18A-AA), arrested at G2, released into MC medium and fixed after 60 min of further incubation and stained for CREST (green), α-tubulin (α-TUB, red) and DNA (blue). Scale bar: 10 μm. Graph: percent of bipolar spindles with misaligned chromosomes (more than three chromosomes falling outside the two internal quarters of the interpolar distance). Around 100 cells were scored in 4 independent microscopy slide fields per sample. Error bars refer to variability within three independent experiments performed under similar experimental conditions. The p-values, reported above the bars, were calculated from comparison of percent of bipolar spindles with misaligned chromosomes of Cdk1-AF cells with that of Cdk1-AF + Kif18A-WT cells (*p* = 0,2360) and Cdk1-AF + Kif18A-AA cells (*p* = 0,0047) in three independent experiments, using a two-tailed unpaired *t*-test. **(B)** Blots: Lysates of HeLa cells co-transfected with a Cdk1-AF expression vector plus an empty vector (Mock) or plus Kif18A-WT or Kif18A-AA expression vectors were probed for the indicated antigens.

It has been shown that the serines 674 and 684 of human Kif18A are major sites of inhibitory phosphorylation by Cdk1, thus, we asked whether expressing a Kif18A mutant version in which S674 and S684 are mutated into non-phosphorylatable alanine (S674A/S684A; Kif18A-AA), could compensate alignment defects in i-Cdk1-downregulated cells. To this end, HeLa cells were co-transfected with Cdk1-AF-expression vector plus either an empty vector or a wild type Kif18A-expression vector (Kif18A-WT) or a S674A/S684A-Kif18A-expression vector (Kif18A-AA), arrested at G2, released into MC medium (as described in Figure 1) and fixed after 60 min of further incubation (Figures 4A, B). Scoring chromosome alignment in cells with bipolar spindles showed that co-expressing Kif18A-WT with Cdk1-AF had some mild effect on chromosome alignment but unable to significantly reduce the overall percent of misaligned spindles relatively to the sole Cdk1-AF expression (p-value = 0.2360; spindles were scored as misaligned when more than three chromosomes were outside the two middle quarters of the interpolar distance; see Material and Methods section; Figure 4A). On the contrary, co-expressing Kif18A-AA with Cdk1-AF had a strong effect in reversing alignment defects, significantly reducing the percent of spindles with misaligned chromosomes (p-value = 0,0047; Figure 4A).

## Discussion

Kif18A has been shown to promote chromosome alignment at the spindle equator by accumulating at the K-MT plus ends, thereby confining centromere movements by suppressing MT dynamics (Risteski et al., 2021; Mayr et al., 2007; Stumpff et al., 2008; Du et al., 2010). However, this action has been shown to be antagonized by direct phosphorylation of Kif18A by Cdk1 (Häfner et al., 2014). Therefore, how Kif18A exerts its action for chromosome alignment in the presence of Cdk1 activity remains unclear. In this study, we investigated whether Kif18A action is regulated by i-Cdk1, a recently identified subpopulation of spindle-localized Cdk1 that is inhibited by phosphorylation during mitosis and is required for spindle assembly (Serpico et al., 2022). In mitotic human cells with reduced levels of i-Cdk1, we observed defective bipolar spindles that were often elongated and with bioriented chromosome pairs that failed to align at the equator of the metaphase plate and that exhibited diminished Kif18A accumulation at their K-MT plus ends (Figures 2 and 3; Supplementary Figures S1, S2). These effects were reversed by mild Cdk1 inhibition, that restored i-Cdk1 as previously shown (Figures 2, 3; Supplementary Figures S1, S2) (Serpico et al., 2022). Through cell fractionation experiments, we observed that at prometaphase, Kif18A was mainly found in the soluble fraction rather than the pellet fraction (Figure 3C). In contrast, at metaphase Kif18A accumulated in the microtubular pellet fraction and appeared as a faster migrating form on SDS/PAGE, compared to its migration from the soluble fraction, indicating dephosphorylation (Figure 3C). The accumulation of Kif18A in the pellet fraction in its faster migrating form was strongly reduced in cells with reduced i-Cdk1 levels, while both distribution and migration were reversed upon re-establishing i-Cdk1 (Figure 3C). Furthermore, the faster migrating form of Kif18A found in the pellet fraction of metaphase cells was shown to interact with i-Cdk1, which was associated with the presumably active form of PP1α, dephosphorylated at the inhibitory T320 (Figure 3D). In addition, the expression of a Cdk1-dependent phosphorylation-resistant mutant of Kif18A alleviated chromosome alignment defects in cells with reduced i-Cdk1 levels (Figure 4). Collectively, our findings suggest a scenario in which the localization of i-Cdk1 along spindle MTs facilitates the local dephosphorylation of Kif18A by PP1, enabling it to regain its ability to concentrate at the plus end of K-MTs, reduce MT dynamics, and enhance chromosome alignment. While loss of Kif18A function seems to be tolerated by euploid cells, it is crucial for the survival of certain aneuploid cancers, revealing its strong potential as an antitumor therapeutic target (Gomes et al., 2022; Sepaniac et al., 2021; Quinton et al., 2021; Cohen-Sharir et al., 2021; Marquis et al., 2021; Payton et al., 2024; Gliech et al., 2024). Moreover, the sensitivity of aneuploid cancer cells to chemical Kif18A inhibition appears to be linked to their capacity to delay mitosis exit through an efficient SAC response (Gliech et al., 2024; Varetti et al., 2011; Serpico and Grieco, 2020). Given that Cdk1 is a key kinase that activates the SAC and that inhibiting Wee1 extends mitosis in a SAC-dependent manner, it can be hypothesized that combining Wee1 and Kif18A inhibition may potentiate therapeutic effectiveness against aneuploid cancers (Visconti et al., 2015; Toledo et al., 2015).

## Data Availability

The original contributions presented in the study are included in the article/Supplementary Material, further inquiries can be directed to the corresponding author.
