# Supplementary material for: Chromosome alignment and Kif18A action rely on spindle-localized control of Cdk1 activity
Source: Front Cell Dev Biol. 2024 Nov 14;12:1490781. doi: 10.3389/fcell.2024.1490781 (PMC11602486; doi:10.3389/fcell.2024.1490781)
Supplement: Supplementary file 1 [file DataSheet1.pdf]

Supplementary Figure 1

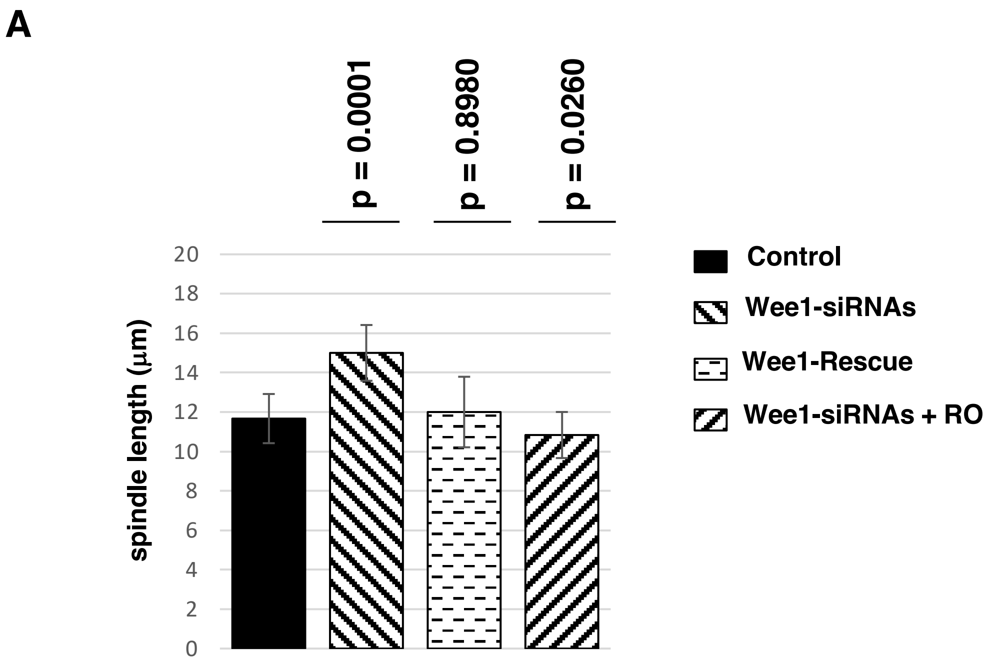

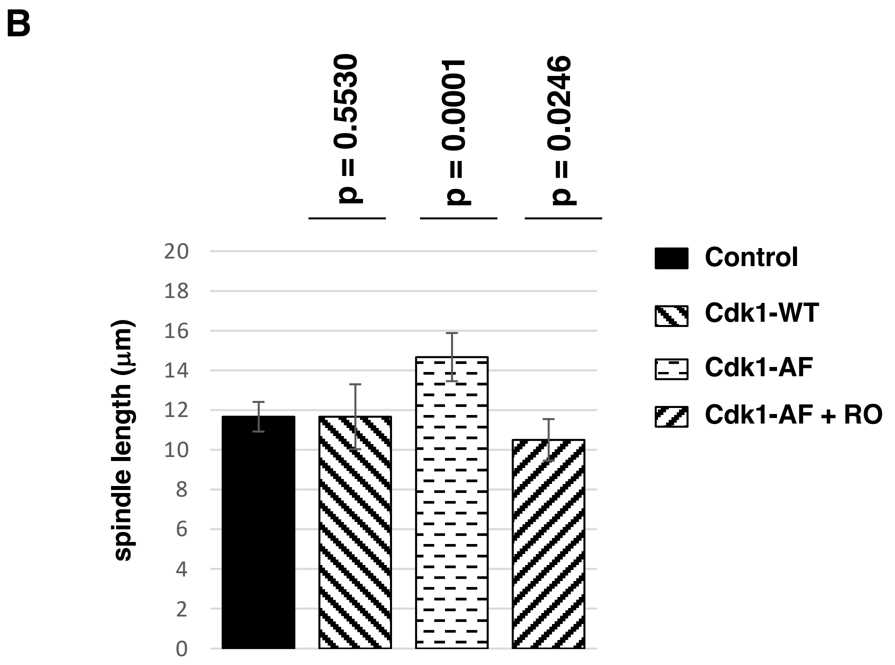

Supplementary Figure 1. Spindle length in cells with reduced i-Cdk1

Graphs showing average spindle length (µm) in (**A**) non targeting-siRNA (Control), Wee1-siRNAs, Wee1-Rescue and Wee1-siRNAs + RO-3306 (0.5 µM; + RO) HeLa cells, treated as described in Figure 1 A and in (**B**) HeLa cells transfected with an empty vector (Control), Cdk1-WT- and Cdk1-AF-expression vectors and in a sample of the Cdk1-AF-transfected cells that was also further treated RO-3306 (0.5 µM; Cdk1-AF + RO), as described in Figure 1 B. Spindle length was scored by measuring the distance from the two opposite α-tubulin apexes; error bars refer to standard deviation of spindle length in 45 bipolar spindles analyzed per condition from three independent experiments (15 spindles analyzed per experiment in each condition). The p-values, reported above the bars, were calculated from comparison of spindle length of the relative Control cells with that of cells after the various treatments.

Supplementary Figure 2

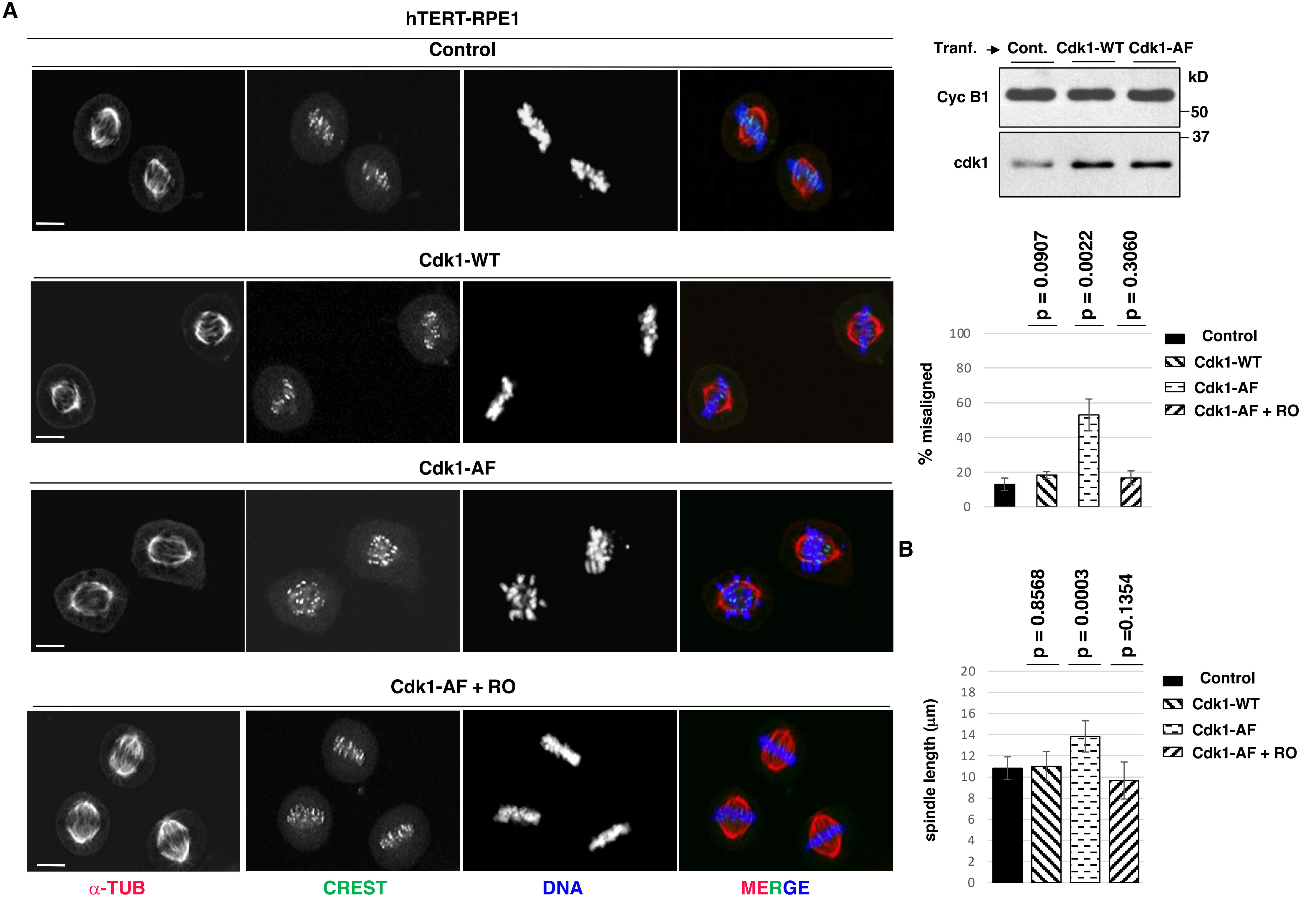

Supplementary Figure 2. Dependence of chromosome alignment on i-Cdk1 in hTERT-RPE1

(**A**) hTERT-RPE1 cells were transfected with an empty vector (Control), with a Cdk1-WT expression vector or with a Cdk1-AF expression vector. 6 hours post transfection, cells were arrested at G2 by addition of RO-3306 (9 μM) for further 16 hours incubation, released into fresh MC medium (see Material and Methods section), fixed after 80 minutes of further incubation and analyzed by immunofluorescence. Cells were stained for DNA (blue), α-tubulin (α-TUB, red) and CREST (green) at 80 min upon release from G2 arrest. A portion of Cdk1-AF-transfected cells received low RO-3306 (0.5 μM) at 60 min (Cdk1-AF + RO); as control vehicle was added (DMSO). Scale bar: 10 μm. Graph: percent of bipolar spindles with misaligned chromosomes in Control, Cdk1-WT, Cdk1-AF, and Cdk1-AF + RO cells. Spindles were scored as with misaligned chromosomes when more than three chromosomes were outside the two internal quarters of the interpolar distance. Around 100 cells were scored in 4 independent microscopy slide fields per sample. Error bars refer to standard deviation of three independent experiments performed under similar experimental conditions. The p-values, reported above the bars, were calculated from comparison of percent of bipolar spindles with misaligned chromosomes of Control cells with that of Cdk1-WT cells (p = 0.0907), Cdk1-AF cells (p = 0,0022) and Cdk1-AF + RO cells (p = 0,3060), in three independent experiments, using a two-tailed unpaired *t*-test. Blots: cell samples from Control, Cdk1-WT and Cdk1-AF were lysed and lysates probed for the indicated antigens. (**B**) Graph showing average spindle length (μm) in hTERT-RPE1 cells treated as described in (**A**). Spindle length was scored by measuring the distance from the two opposite α-tubulin apexes; error bars refer to standard deviation of spindle length in 45 bipolar spindles analyzed per condition from three independent experiments (15 spindles analyzed per experiment in each condition). The p-values, reported above the bars, were calculated from comparison of spindle length of Control cells with spindle length of Cdk1-WT-, Cdk1-AF and Cdk1-AF + RO cells.

Supplementary Figure 3

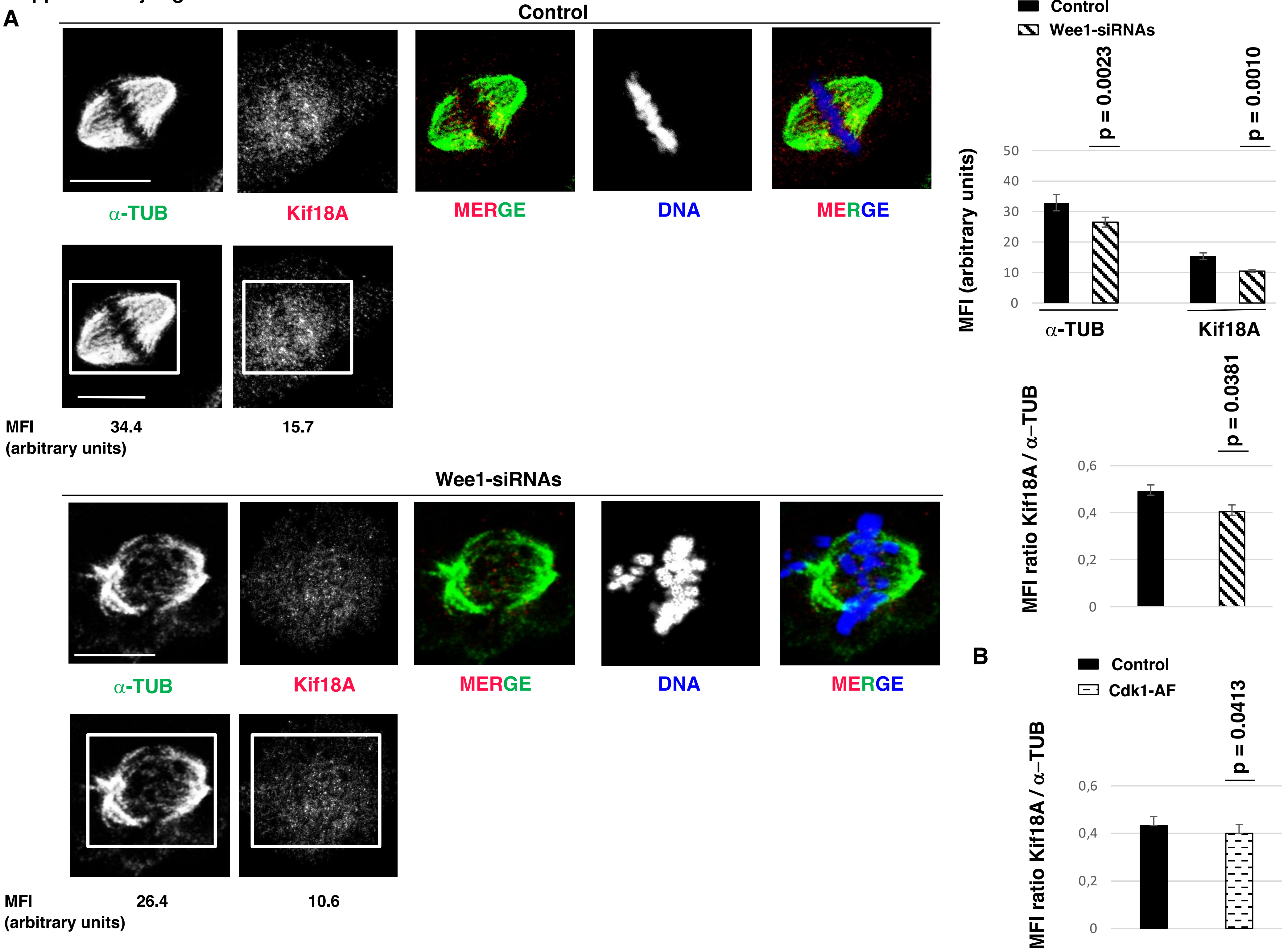

Supplementary Figure 3. Analysis of Kif18A and α-tubulin signals in cells with reduced i-Cdk1

(**A**) Control and Wee1-siRNAs cells were treated as described in Figure 1 A. Cells were stained for DNA (blue), α-tubulin (α-TUB, green) and Kif18A (red) and the mean fluorescence intensity (MFI) of the Kif18A and α-tubulin signals, in the spindle area (an example is shown in rectangles in the lower images of Kif18A and α-tubulin), was quantified through ImageJ software and indicated (as arbitrary units; scale bar: 10 μm). The top graph indicates the average mean fluorescence intensity (MFI) of α-tubulin (α-TUB) and Kif18A signals in Control and Wee1-siRNAs cells, from 20 bipolar spindle per condition, from 2 independent experiments (total of 40 spindle analyzed per condition). The lower graph indicates the ratio of Kif18A / α-tubulin MFI (from 20 bipolar spindle per condition from 2 independent experiments; total of 40 spindle analyzed per condition). Error bars indicate standard deviation. The p-values, reported above the bars, were calculated from comparison of the average α-tubulin (α-TUB) and Kif18A MFI (top graph) and the ratio Kif18A / α-tubulin MFI of Control cells with Wee-siRNA cells (lower graph; total of 40 spindle analyzed per condition). (**B**) The graph indicates the ratio of Kif18A / α-tubulin MFI in Control and Cdk1-AF cells, treated as described in Figure 1 B, in 20 bipolar spindles per condition from 2 independent experiments (total of 40 spindles analyzed per condition). Error bars indicate standard deviation. The p-value, reported above the bar, was calculated from comparison of the ratio of Kif18A / α-tubulin MFI (total of 40 spindle analyzed per condition) of Control cells with that of Cdk1-AF cells.
